# *Pou4f1* and *Pou4f2* Are Dispensable for the Long-Term Survival of Adult Retinal Ganglion Cells in Mice

**DOI:** 10.1371/journal.pone.0094173

**Published:** 2014-04-15

**Authors:** Liang Huang, Fang Hu, Xiaoling Xie, Jeffery Harder, Kimberly Fernandes, Xiang-yun Zeng, Richard Libby, Lin Gan

**Affiliations:** 1 Flaum Eye Institute and Department of Ophthalmology, University of Rochester School of Medicine and Dentistry, Rochester, New York, United States of America; 2 Department of Ophthalmology, First Affiliated Hospital of Gannan Medical University, Ganzhou, Jiangxi, China; 3 College of Life and Environmental Sciences, Hangzhou Normal University, Hangzhou, Zhejiang, China; Columbia University, United States of America

## Abstract

**Purpose:**

To investigate the role of *Pou4f1* and *Pou4f2* in the survival of adult retinal ganglion cells (RGCs).

**Methods:**

Conditional alleles of *Pou4f1* and *Pou4f2* were generated (*Pou4f1^loxP^* and *Pou4f2^loxP^* respectively) for the removal of *Pou4f1* and *Pou4f2* in adult retinas. A tamoxifen-inducible Cre was used to delete *Pou4f1* and *Pou4f2* in adult mice and retinal sections and flat mounts were subjected to immunohistochemistry to confirm the deletion of both alleles and to quantify the changes in the number of RGCs and other retinal neurons. To determine the effect of loss of *Pou4f1* and *Pou4f2* on RGC survival after axonal injury, controlled optic nerve crush (CONC) was performed and RGC death was assessed.

**Results:**

*Pou4f1* and *Pou4f2* were ablated two weeks after tamoxifen treatment. Retinal interneurons and Müller glial cells are not affected by the ablation of *Pou4f1* or *Pou4f2* or both. Although the deletion of both *Pou4f1* and *Pou4f2* slightly delays the death of RGCs at 3 days post-CONC in adult mice, it does not affect the cell death progress afterwards. Moreoever, deletion of *Pou4f1* or *Pou4f2* or both has no impact on the long-term viability of RGCs at up to 6 months post-tamoxifen treatment.

**Conclusion:**

*Pou4f1* and *Pou4f2* are involved in the acute response to damage to RGCs but are dispensable for the long-term survival of adult RGC in mice.

## Introduction

Glaucoma, a retinal degeneration disease characterized by progressive loss of retinal ganglion cells (RGCs), affected over 60 million people worldwide in 2010 and the number will increase to about 80 million by 2020 [1]. As the second leading cause of blindness, glaucoma is responsible for millions of blindness worldwide and most clinical glaucoma cases are in advanced conditions due to the inconspicuous early symptoms and the lack of effective early diagnosis. Understanding of the molecular mechanism underlying glaucomatous optic neuropathy is crucial for the diagnosis and treatment of glaucoma. The three closely related Class IV POU-homeodomain (POU4F) transcription factors, POU4F1, POU4F2 and POU4F3, are expressed in developing and adult RGCs, and are key components of a regulatory cascade of RGC development and survival [2], [3]. During retinal development, *Pou4f2* expression starts in more than 80% RGC precursors at embryonic day 11.5 (E11.5), a time when RGCs are first generated [2], [4]. Afterwards, *Pou4f1* and *Pou4f3* are expressed in 80% and 20% developing RGCs, respectively [2], [5], [6]. Targeted deletion of *Pou4f2* leads to a loss of about 80% RGCs accompanied by severe axonal defects and abnormal visual driven behavior [4], [7]–[12]. *Pou4f1* has been shown to control the dendritic stratification pattern of selective RGCs, loss of *Pou4f1* resulted in the alteration of dendritic stratification and the ratio of monostratified:bistratified RGCs [12]. Although the deletion of *Pou4f3* alone does not affect the generation and survival of RGCs, *Pou4f2*/*Pou4f3* compound mutant exhibited more severe RGC loss than *Pou4f2* mutant, suggesting a redundant role of *Pou4f3* in regulating the survival of RGCs [13]. Thus each POU4F gene plays a distinctive role in RGC development and survival. But whether POU4F factors are required for the survival of adult RGC remains unknown.

Previous studies have revealed that similar to other neuronal degeneration diseases, the progressive death of RGCs in glaucoma is through apoptosis pathway [14]–[19] mediated by the BCL2 family proteins [20]. BAX, the pro-apoptotic BCL2 member required for the normal death of RGC during development [21], [22], has been identified as a major mediator of RGC death in glaucoma [14], [23], [24]. Deficiency of BAX gives long-term protection of RGC soma and slows axonal loss in glaucoma mouse models [14], [23]. On the other hand, the pro-survival factors of BCL2 family, such as BCL2 and BCL-X, promote cell survival by preventing the activation of their pro-apoptotic relatives [25]–[29]. Previous evidences have stated that POU4F1 promotes the expression of BCL2 pro-survival gene and suppresses BAX activation to protect neurons from programmed cell death [30]–[32]. Meanwhile, overexpression of pro-survival factors BCL-X and BCL2 protects RGCs from death during development and after axonal injury in the adult [18], [33]–[35]. Interestingly, optic nerve crush leads to a rapid decrease in the expression of POU4F proteins in rat RGCs [36]. Therefore, it is conceivable that loss of POU4F factors could result in the progressive degeneration of adult RGCs and render RGCs more sensitive to the optic nerve injury and accelerate the apoptosis of RGCs.

In order to investigate the role of POU4F factors in adult RGCs, we focused on POU4F1 and POU4F2 whose combined expression covers almost all RGCs in the adult retina. We generated *Pou4f1* and *Pou4f2* conditional null alleles (*Pou4f1^loxP/loxP^* and *Pou4f2^loxP/loxP^*) and used tamoxifen-inducible CreER to inactivate *Pou4f1* or *Pou4f2* or both in adult mice. We showed that *Pou4f1* and *Pou4f2* were effectively deleted two weeks after tamoxifen treatment. Further analysis of RGCs in retinal section and flat mount samples surprisingly revealed that deletion of *Pou4f1* or *Pou4f2* or both had no effect on the total number of RGCs at test timepoints from two weeks to six months after tamoxifen treatment. Furthermore, examination of RGCs in controlled optic nerve crush of *Pou4f1*/*Pou4f2* compound null mice also revealed that deletion of *Pou4f1* and *Pou4f2* did not accelerate the apoptosis of RGCs. Therefore, our results strongly argue for the dispensable role of *Pou4f1* and *Pou4f2* in regulating the survival of RGCs in adult mice.

## Results

### Conditional Deletion of *Pou4f1*, *Pou4f2*, or Both in Adult Mice

**Figure 1:**
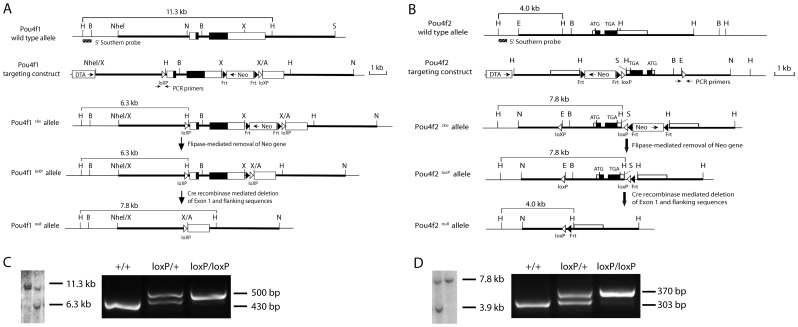
Generation of *Pou4f1* and *Pou4f2* conditional knockout alleles. (A) Targeting strategy for generating *Pou4f1* conditional null allele. (B) Targeting strategy for generating *Pou4f2* conditional null allele. (C) Southern blot confirmed selection of *Pou4f1^cko^* allele and PCR genotyping of *Pou4f1^loxP^* heterozygous and homozygous. (D) Southern blot confirmed selection of *Pou4f2^cko^* allele and PCR genotyping of *Pou4f2^loxP^* heterozygous and homozygous. White boxes are the non-coding exon sequences and black boxes are the coding sequences. Thick bars are the sequences used to generate the homologous arms in the targeting vector. Restriction enzyme sites: A, *AvrII*; B, *BamHI*; E, *EcoRI*; H, *HindIII*; N, *NotI*; S, *SacII*; X, *XbaI*.

To assess the function of *Pou4f1* and *Pou4f2*, we generated *Pou4f1* and *Pou4f2* conditional knockout alleles, *Pou4f1^loxP^* and *Pou4f2^loxP^* (Fig. 1). The *Pou4f1^loxP^* heterozygotes (*Pou4f1^loxP/+^*) and heterozygotes (*Pou4f1^loxP/loxP^*), and the *Pou4f2^loxP^* heterozygotes (*Pou4f2^loxP/+^*) and heterozygotes (*Pou4f2^loxP/loxP^*) appeared normal and were fertile. We further crossed *Pou4f1^loxP^* and *Pou4f2^loxP^* mice with CreER mice to generate *Pou4f1CKO* (*Pou4f1^loxP/loxP^; CreER*), *Pou4f2CKO* (*Pou4f2^loxP/loxP^; CreER*), and *DoubleCKO* (*Pou4f1^loxP/loxP^; Pou4f2^loxP/loxP^; CreER*) conditional knockout mice, respectively.

**Figure 2:**
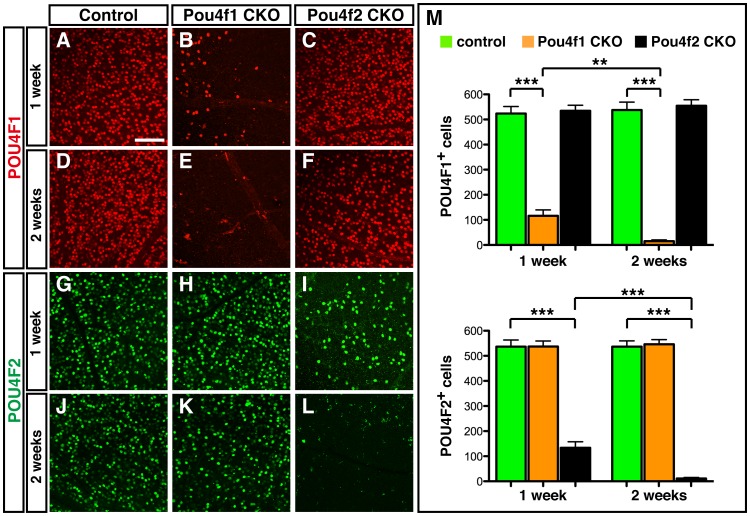
Deletion of *Pou4f1* and *Pou4f2* after tamoxifen treatment. Flat mounted retinas from *Pou4f1CKO* and *Pou4f2CKO* mice were collected one week and two weeks after tamoxifen treatment respectively. (A–F) Immunostaining of anti-POU4F1 in control (A, D), *Pou4f1CKO* (B, E) and *Pou4f2CKO* (C, F) at one week (A–C) and two weeks after (D–F) tamoxifen treatment. (G–L) Immunostaining of anti-POU4F2 in control (G, J), *Pou4f1CKO* (H, K) and *Pou4f2CKO* (I, L) at one week (G–I) and two weeks after (J–L) tamoxifen treatment. (M) Quantification of POU4F1^+^ and POU4F2^+^ cells in 40X field (equals to 1,600 µm^2^). Scale bar equals to 100 µm.

In order to investigate the role of *Pou4f1* and *Pou4f2* in adult RGCs, we deleted *Pou4f1* and *Pou4f2* by intraperitoneal injection of tamoxifen at P30 at the dosage of 5 mg/40 g bodyweight for five consecutive days. Their control *Pou4f1^loxP/^* ^loxP^ and *Pou4f2^loxP/loxP^* littermates were given oil vehicle only. We collected the retinas from *Pou4f1CKO*, *Pou4f2CKO*, and control mice at one week and two weeks after injection and performed retinal whole mount immunolabeling experiments to evaluate the deletion efficiency (Fig. 2). Immunostaining with anti-POU4F1 and anti-POU4F2 in *Pou4f1CKO* mice revealed that one week after injection, about 20% POU4F1^+^ RGCs were left (Fig. 2B and M) compare to the control (Fig. 2A). At two weeks after injection, very few POU4F1^+^ remained, indicating a nearly complete deletion of *Pou4f1* in retina (Fig. 2E and M). Similarly, in *Pou4f2CKO* mice, POU4F2 was expressed in about 28% RGCs one week after tamoxifen treatment (Fig. 2I and M) and in a very few RGCs two weeks after treatment (Fig. 2L and M). Interestingly, deletion of *Pou4f1* in adult RGCs did not affect the expression of *Pou4f2* and vice versa (Fig. 2C, F, H, K, and M).

### Deletion of *Pou4f1* or *Pou4f2* or Both does not Affect Retinal Interneurons and Müller Glial Cells

**Figure 3:**
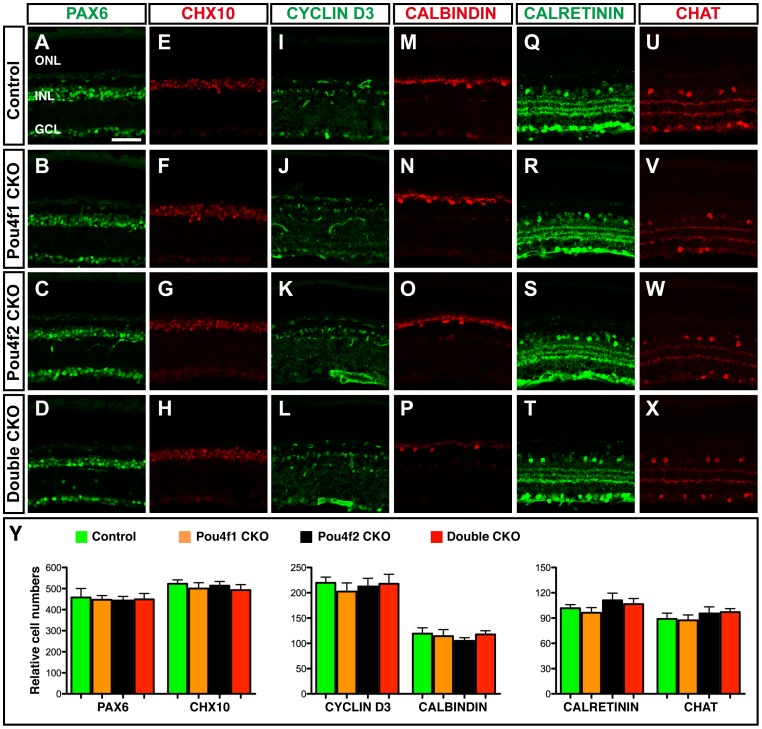
Deletion of *Pou4f1* and/or *Pou4f2* did not affect neurons other than RGC. Retina sections from *Pou4f1CKO*, *Pou4f2CKO, DoubleCKO* and control mice were collected two weeks after tamoxifen treatment. (A–D) PAX6^+^ pan-amacrine cells (green) in Control mice (A), *Pou4f1CKO* (B), *Pou4f2CKO* (C) and *DoubleCKO* mice (D). (E–H) CHX10^+^ pan-bipolar cells (red) in Control mice (E), *Pou4f1CKO* (F), *Pou4f2CKO* (G) and *DoubleCKO* mice (H). (I–L) CYCLIN D3^+^ Müller glial cells (green) in Control mice (I), *Pou4f1CKO* (J), *Pou4f2CKO* (K) and *DoubleCKO* mice (L). (M–P) CALBINDIN^+^ horizontal cells (red) in Control mice (M), *Pou4f1CKO* (N), *Pou4f2CKO* (O) and *DoubleCKO* mice (P). (Q–T) Three CALRETININ^+^ bands within IPL (green) are persisted in control mice (Q), *Pou4f1CKO* (R), *Pou4f2CKO* (S) and *DoubleCKO* mice (T). (U–X) Two CHAT^+^ bands within IPL (red) are persisted in control mice (U), *Pou4f1CKO* (V), *Pou4f2CKO* (W) and *DoubleCKO* mice (X). (Y) Quantification of cell number of each marker per section. Cell numbers of PAX6 and CHX10 positive cells are relative numbers per 1,000 µm. Abbreviations: GCL, ganglion cell layer; IPL, inner plexiform layer; INL, inner nuclear layer; OPL, outer plexiform layer; ONL, outer nuclear layer. Scale bar equals to 100 µm.

**Figure 4:**
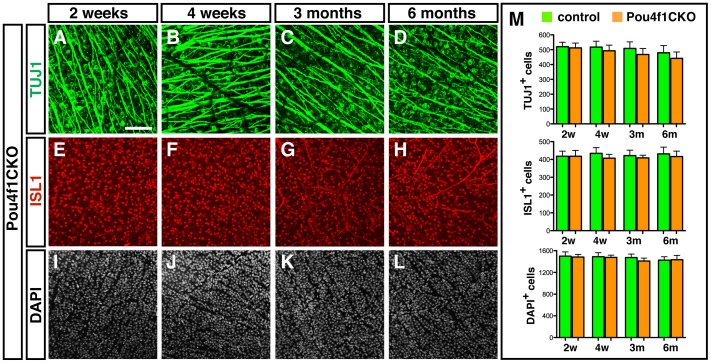
No significant change in the number of RGC in adult *Pou4f1CKO* mice. Retina flat mounts of control and *Pou4f1CKO* mice were collected after tamoxifen injection. (A–C) Two weeks after tamoxifen treatment, RGCs labeled by TUJ1 (A) and ISL1 (B) and DAPI in GCL (C); (D–F) Four weeks after tamoxifen treatment, RGCs labeled by TUJ1 (D) and ISL1 (E) and DAPI in GCL (F); (G–I) Three months after tamoxifen treatment, RGCs labeled by TUJ1 (G) and ISL1 (H) and DAPI in GCL (I); (J–L) Six months after tamoxifen treatment, RGCs labeled by TUJ1 (J) and ISL1 (K) and DAPI in GCL (L); (M) Quantification results of each cell marker in 1,600 µm^2^. Scale bar equals to 100 µm.

Since *Pou4f1* and *Pou4f2* are only expressed within RGCs in the retina, deletion of *Pou4f1* or *Pou4f2* or both is not expected to affect other retinal cells. To confirm this, we assessed the change in non-RGC cells in *Pou4f1CKO*, *Pou4f2CKO* and *DoubleCKO* retinas two weeks after tamoxifen treatment (Fig. 3). As expected immunolabeling with retinal cell markers revealed no significant change in the number of PAX6^+^ amacrine cells (Fig. 3A–D, Y), CHX10^+^ bipolar cells (Fig. 3E–H, Y), calbindin^+^ horizontal cells (Fig. 3I–L, Y) and cyclin D3^+^ Müller glial cells (Fig. 3M–P, Y) when *Pou4f1* or *Pou4f2* or both were deleted. Additionally, immunolabeling with anti-calretinin and anti-CHAT revealed that the number of these amacrine subtypes and their dendritic stratification in the inner plexiform layer (IPL) were unaffected (Fig. 4Q–Y). Thus, loss of *Pou4f1* and *Pou4f2* does not appear to affect retinal interneurons and Müller glial cells.

### Deletion of *Pou4f1* or *Pou4f2* or Both does not Affect the Survival of Adult RGCs Under Normal Conditions

**Figure 5:**
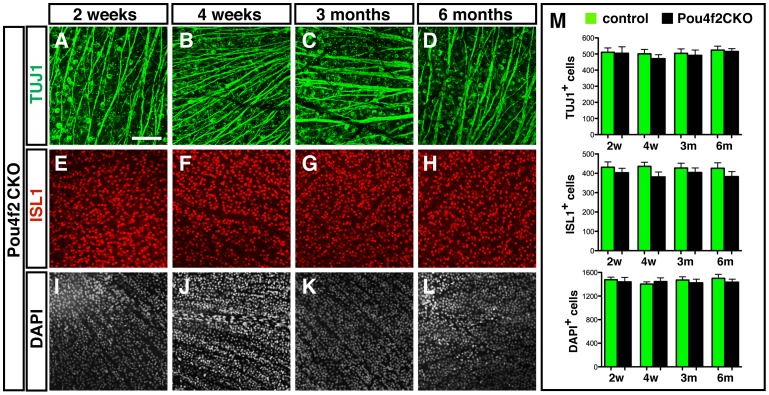
No significant change in the number of RGCs in adult *Pou4f2CKO* mice. Retina flat mounts of control and *Pou4f2CKO* mice were collected after tamoxifen injection. (A–C) Two weeks after tamoxifen treatment, RGCs labeled by TUJ1 (A) and ISL1 (B) and DAPI in GCL (C); (D–F) Four weeks after tamoxifen treatment, RGCs labeled by TUJ1 (D) and ISL1 (E) and DAPI in GCL (F); (G–I) Three months after tamoxifen treatment, RGCs labeled by TUJ1 (G) and ISL1 (H) and DAPI in GCL (I); (J–L) Six months after tamoxifen treatment, RGCs labeled by TUJ1 (J) and ISL1 (K) and DAPI in GCL (L); (M) Quantification results of each cell marker in 1,600 µm^2^. Scale bar equals to 100 µm.

To investigate the role of *Pou4f1* in adult RGCs, we collected retinas from *Pou4f1CKO* and control mice at two weeks, four weeks, three months and six months after tamoxifen treatment respectively. RGC markers anti-TUJ1 and anti-ISL1 were used to label RGCs in flat mounted retina and DAPI was used to label all cells in the GCL (Fig. 4). After quantification of each cell marker, we found that there was no significant change in the number of RGCs labeled for TUJ1 (Fig. 4A–D, M) and ISL1 (Fig. 5E–H, M). In addition, the total number of DAPI^+^ cells in GCL remained unchanged (Fig. 5I–L, M). Taken together, our results suggest that the deletion of *Pou4f1* alone in adult mice did not affect the survival of RGCs.

Similarly, we examined the role of *Pou4f2* in adult RGCs in *Pou4f2CKO* and control mice retinas at two weeks, four weeks, three months and six months after tamoxifen treatment (Fig. 5). Quantification of each cell marker revealed that the number of TUJ1^+^ RGCs (Fig. 5A–D, M), ISL1^+^ RGCs (Fig. 5E–H, M) and DAPI^+^ cells in GCL (Fig. 5I–L, M) were similar between *Pou4f2*-null and control mice, indicating that the deletion of *Pou4f2* in adult mice did not influence the survival of RGCs.

**Figure 6:**
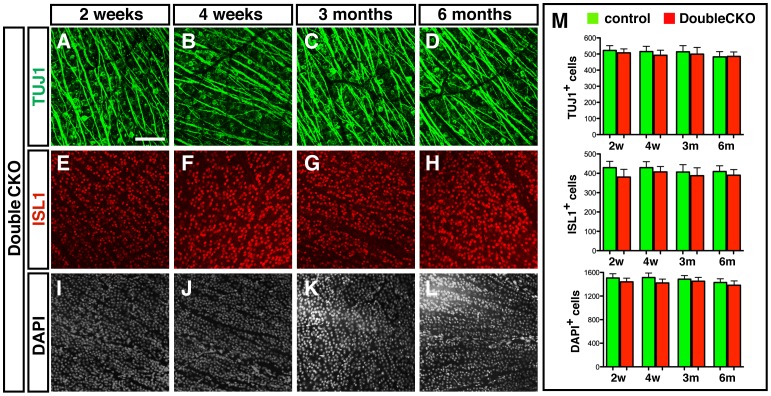
No significant change in the number of RGCs in adult *DoubleCKO* mice. Retina flat mounts of control and *DoubleCKO* mice were collected after tamoxifen injection. (A–C) Two weeks after tamoxifen treatment, RGCs labeled by TUJ1 (A) and ISL1 (B) and DAPI in GCL (C); (D–F) Four weeks after tamoxifen treatment, RGCs labeled by TUJ1 (D) and ISL1 (E) and DAPI in GCL (F); (G–I) Three months after tamoxifen treatment, RGCs labeled by TUJ1 (G) and ISL1 (H) and DAPI in GCL (I); (J–L) Six months after tamoxifen treatment, RGCs labeled by TUJ1 (J) and ISL1 (K) and DAPI in GCL (L); (M) Quantification results of each cell marker in 1,600 µm^2^. Scale bar equals to 100 µm.

Previous studies have shown that POU4F transcription factors are redundantly required for the differentiation and survival of RGCs during development [5]. Therefore, we sought to test whether the absence of RGC death in *Pou4f1*CKO or *Pou4f2*CKO mice was due to the overlapping expression of *Pou4f1* and *Pou4f2* in a majority of the RGCs. We generated the *Pou4f1/Pou4f2 DoubleCKO* mice and used the same strategy to label the RGCs in the GCL (Fig. 6). After quantification, we found that deletion of *Pou4f1* and *Pou4f2* did not impact the number of TUJ1^+^ RGCs (Fig. 6A–D, M) or ISL1^+^ RGCs (Fig. 6E–H, M) at two weeks to 6 months post tamoxifen treatment. Nor did it affect the total number of cells labeled by DAPI in the GCL (Fig. 6I–L, M).

**Figure 7:**
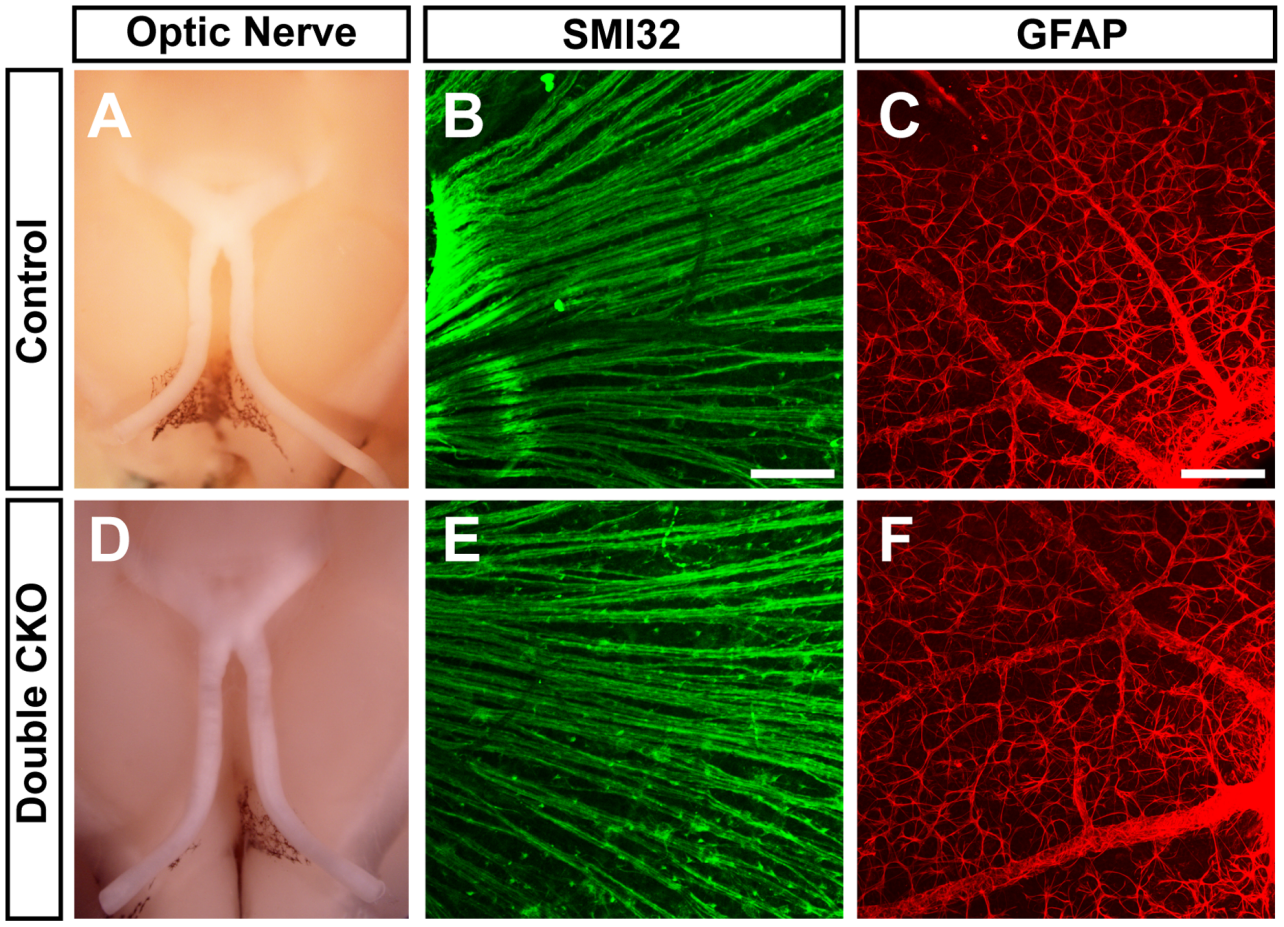
No significant change in optic nerve diameter, axonal elongation and glia activation in control and doubleCKO mice. Both control and doubleCKO mice were collected six months after tamoxifen treatment. No significant change in the optic nerve diameter (A and D), SMI32 immunolabeling (B and E) and GFAP immunolabeling (C and F) is seen between control and doubleCKO mice. Scale bar equals to 100 µm.

To analyze the effect of *Pou4f1* and *Pou4f2* deletion on RGC axonal elongation, we dissected optic nerve from control and doubleCKO mice six months after tamoxifen treatment, and observed that the optic nerves in the control and doubleCKO mice appeared similar in size (Fig. 7A, D). Furthermore, SMI32 immunstaining of the retinal wholemounts revealed similar RGC axon bundles in the neural fiber layer in the control and doubleCKO retinas (Fig. 7B, E). GFAP immunolabeling (Fig. 7C, F) also revealed no difference in glial activation in both control and doubleCKO mice. Thus, our results suggest that *Pou4f1* and *Pou4f2* are dispensable in adult RGCs under normal conditions.

### Loss of *Pou4f1* and *Pou4f2* delays the RGC Apoptosis in the Mouse Controlled Optic Nerve Crush Model

**Figure 8:**
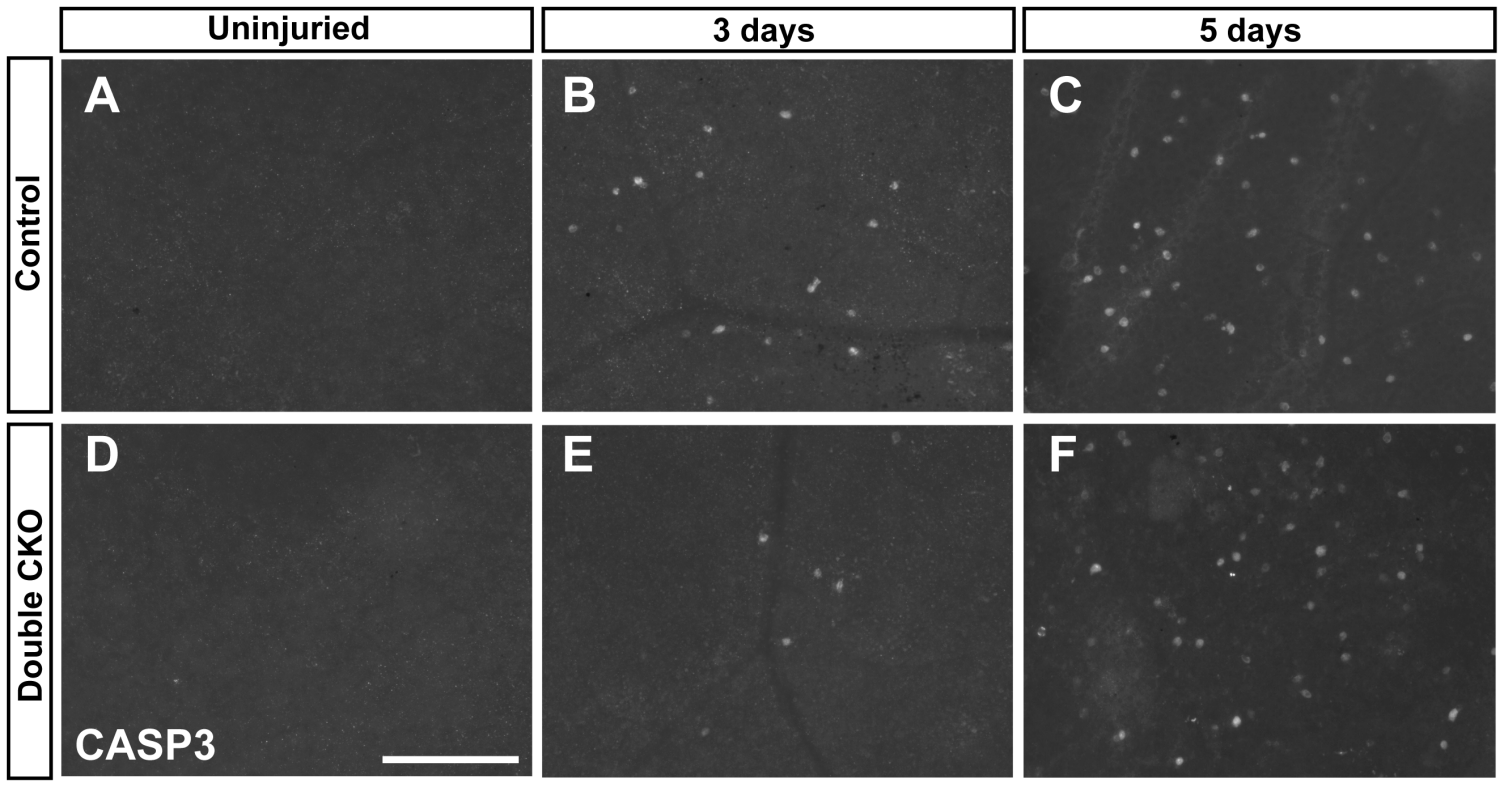
Delayed RGC death in doubleCKO mice at early time point after CONC. CONC was performed in both control and doubleCKO mice one month after tamoxifen treatment. (A, D) Absence of apoptosis in the uninjured retinas. (B, E) 3 days after CONC, RGC death is delayed in doubleCKO mice. (C, F) No significant difference in the number of apoptosis cells in control and doubleCKO retinas 5 days after CONC. Scale bar equals to 100 µm.

Though our above results demonstrate that *Pou4f1* and *Pou4f2* are not essential for the survival of adult RGCs under normal conditions, it is possible that loss of *Pou4f1* and *Pou4f2* might render RGCs more susceptible to cell death under stresses such as upon optic nerve injury. To test this, we performed controlled optic nerve crush (CONC) in the *DoubleCKO* and control mice four weeks after tamoxifen treatment and analyzed the number of apoptotic cells labeled by anti-activated caspase 3 in the GCL of flat mount retinas (Fig. 8). At 3 days after CONC, there was a significant difference between control and the *DoubleCKO* mice (Fig. 8B, E). However, contrary to the accelerated cell death in RGC development, loss of *Pou4f1* and *Pou4f2* in the *DoubleCKO* mice delayed the death of RGCs in the CONC retinas initially. The protective effect by the loss of *Pou4f1* and *Pou4f2* was temporary and was waning at 5 days after CONC, when the difference in apoptotic cells became insignificant (Fig. 8 C, F and Table 1).

**Table 1:** Cell counts of caspase3^+^ cells after CONC in *DoubleCKO* and control retinas.

		Mean ± SD	Number of animals	% control	P vaule
3 Days	Control	89±39	6	100	0.019
	DoubleCKO	40±16	6	45	
5 Days	Control	276±33	3	100	0.52
	DoubleCKO	255±41	3	92	

## Discussion

POU4F family members are crucial factors controlling the development and survival of a variety of neurons in both central and peripheral nervous systems. Deletion of each *Pou4f* gene results in the neuronal death phenotypes during the development of different organs [4], [9], [37]–[40]. POU4F family has also been associated with human degeneration disease, such as the progressive hearing loss caused by an 8-base pair deletion in human *POU4F3* resulting a truncated protein [37]. Mutant POU4F3 loses most of its transcriptional activity and ability to bind to DNA in a nondominant-negative manner [41]. In the retina, all three POU4F members are expressed only in RGCs. Based on their continuous expression in adult RGCs and the similarity in structure and function of all POU4F members, we hypothesized that like the mutation of POU4F3 results in the hearing loss [37], loss of POU4F function may affect the survival of adult RGCs. Since POU4F proteins promote neuronal survival by activating the pro-survival genes and inhibiting the pro-death genes [31], [32], [42]–[44], POU4F proteins are the candidates to rescue RGC from death in glaucoma.

In our study, we investigate the role of *Pou4f1* and *Pou4f2* in adult RGCs by generating the novel *Pou4f1CKO*, *Pou4f2CKO* and *DoubleCKO* mouse models, in which the expression of *Pou4f1*, *Pou4f2* and both can be deleted in adult by tamoxifen-inducible Cre recombinase. Expression of *Pou4f1* and *Pou4f2* are significantly reduced one week after tamoxifen treatment and are ablated two weeks after treatment (Fig. 2). Strikingly, deletion of *Pou4f1* or *Pou4f2* or both does not affect the number of RGCs in retina at two weeks to six months after tamoxifen treatment (Fig. 4–6). In addition, although the apoptosis of RGCs seems delayed in *DoubleCKO* mice at three days after CONC, there is no significant difference in five days after CONC (Table 1). All these results suggest that unlike developing RGCs during embryogenesis, the survival of adult RGCs in normal and CONC retinas might not require *Pou4f1* and *Pou4f2* or at least not *Pou4f1* and *Pou4f2* alone. During RGC development, *Pou4f2* is expressed in the ganglion cell precursors and is required for the normal differentiation and axon pathfinding of RGCs and for the expression of *Pou4f1*. Targeted mutation of *Pou4f2* enhanced apoptosis of RGCs and resulted in a loss of 70% RGCs. However, in our experiments, deletion of *Pou4f2* does not affect the survival of RGCs, suggesting that the role of *Pou4f2* in RGCs survival might be restricted only in developing RGCs but not the mature RGCs. Among other possible factors that could compensate for the loss of POU4F1 and POU4F2 function, POU4F3 plays an essential role in the survival of RGCs during development [13]. In adult mice, the expression of POU4F3 remains in RGCs and could play a role in the survival of adult RGCs. However, unlike the expression of POU4F1 and POU4F2 in most RGCs, POU4F3 is expressed in far fewer RGCs [2]. Thus, it is unlikely that POU4F3 could substitute for POU4F1 and POU4F2 in *Pou4f1CKO*, *Pou4f2CKO* and *DoubleCKO* mice. The LIM-homeodomain transcription factor ISL1 is expressed in developing RGCs and functions synergistically with POU4F to regulate the development and survival of RGCs during embryogenesis [38]. In adults, ISL1 expression persists in RGCs, starburst amacrine cells, and ON-bipolar cells. It would be interesting to test if ISL1 could compensate for the loss of POU4F1 and POU4F2 in adult retinas using triple conditional knockout of *Pou4f1*, *Pou4f2* and *Isl1.*

Overall, our results imply that the survival mechanism of RGCs differs in adults from developmental stages. During neurodevelopment, the three members of the POU4F subfamily transcription factors are broadly expressed, either overlappingly or singularly, in a variety of nervous systems and are essential for the development and survival of neurons. The *Pou4f1* knockout mice die at birth due to the severe defects in dorsal root ganglion (DRG), trigeminal ganglion (TG) and selective nucleus in brain [39], [40]. Thus though *Pou4f1* has a crucial role in neuron survival, axonal projection and subtype specification during development of both central and peripheral nervous system [40], [45]–[51], its function in adult neurons remains unknown. Our *Pou4f1CKO* mice provided a new platform to study the role of *Pou4f1* after birth and both *Pou4f1CKO* and *Pou4f2CKO* mice could be the powerful tools for the investigation on the function of *Pou4f1* and *Pouf42* in both development and adult stages.

## Methods

### Animals

To generate the *Pou4f1* conditional knockout (*Pou4f1^cko^*) mice (Fig. 1A), a 7.7 kb NheI-XbaI fragment containing the complete coding sequences was used as the 5′ homologous arm and was subcloned at the XbaI site of the cloning vector. A loxP site was inserted into 5′ of the first exon and a Frt-loxP-flanked Neomycin (Neo) cassette was inserted downstream of the coding sequence. A diphtheria toxin A (DTA) cassette was inserted upstream of 5′ homologous arms and a 5 kb 3′ arm XbaI-SacII fragment was subcloned into the AvrII and NotI sites of the vector. After homologous recombination in mouse embryonic stem (ES) cells, neomycin-resistant clones were picked and conformed by Southern blot (Fig. 1C). Targeted ES cells were then injected into mouse blastocysts to obtain chimeras. Chimeras were bred with wild-type C57BL/6J mice to generate *Pou4f1^cko^* mice. *Pou4f1^cko^* mice were then crossed with Flippase mice (The Jackson Laboratory, Stock# 009086) to remove Neo cassette and to generate the *Pou4f1^cko^* mice.

To generate the *Pou4f2* conditional knockout (*Pou4f2^cko^*) mice (Fig. 1B), we used a 6.5 kb EcoRI-HindIII fragment containing the entire coding sequences as the 5′ homologous arm and subcloned it into the NotI and SalI sites of the cloning vector. A loxP site was inserted 5′ of the first exon. Frt-loxP-flanked Neo cassette and DTA cassette were inserted in the vector for positive and negative selections. A 4.3 kb HindIII fragment as the 3′ homologous arm was placed at the HindIII site of the vector. Similar to making *Pou4f1^cko^* mice, targeted *Pou4f2^cko^* ES cell clones were obtained and confirmed by Southern blot (Fig. 1D), and *Pou4f2^cko^* mice were generated. *Pou4f2^loxP^* mice were obtained by breeding *Pou4f2^cko^* mice with mice expressing Flippase.

This study was carried out in strict accordance with the recommendations in the Guide for the Care and Use of Laboratory Animals of the National Institutes of Health. The animal protocol was approved by the University Committee of Animal Resources (UCAR Protocol No. 101414) at the University of Rochester. All surgery was performed under sodium pentobarbital anesthesia, and all efforts were made to minimize suffering. Embryos were designated as E0.5 at noon on the day at which vaginal plugs were observed. The day of birth was considered as P0.

### Histochemistry and Immunohistochemistry

Staged mouse embryos were dissected and immediately fixed in 4% paraformaldehyde (PFA) in PBS at 4°C for 2–3 hours. Samples were embedded and frozen in OCT medium (Tissue-Tek) after dehydration in graded sucrose and sectioned at 14 µm thickness. Before adult retina samples were harvested, vascular perfusion was performed to eliminate the blood remain in the retinal vessels, and then retinas were dissected and fixed in 4%PFA. Retinal flat mount immunostaining was performed as previously described [52]. Dilution and sources of antibodies used in this study were: mouse anti-POU4F1 (1∶500, Santa Cruz), goat anti-POU4F2 (1∶500, Santa Cruz), mouse anti-Calbindin (1∶5000, Sigma), rabbit anti-Calretinin (1∶2000, Oncogene), rabbit anti-CHAT (1∶200, Chemicon), sheep anti-CHX10 (1∶200, Exalpha), mouse anti-PAX6 (1∶200, DSHB). Mouse anti-Cyclin D3 (1∶100, Santa Cruz). Alexa-conjugated secondary antibodies (Molecular Probes) were used at a dilution of 1∶1,000. Images were captured with a Zeiss 510 META confocal microscope.

### Statistical Analysis

Cell number quantification of different retina cell markers was performed with retina sections and flat mounts from at least three age-matched animals for each cell type. Data are represented as mean±SEM. Statistical analysis was performed using paired two-sample Student’s *t-*test. A value of P<0.05 was considered statistically significant.
